# Age-Related Differences in the Expression of Most Relevant Mediators of SARS-CoV-2 Infection in Human Respiratory and Gastrointestinal Tract

**DOI:** 10.3389/fped.2021.697390

**Published:** 2021-07-28

**Authors:** Roberto Berni Canani, Marika Comegna, Lorella Paparo, Gustavo Cernera, Cristina Bruno, Caterina Strisciuglio, Immacolata Zollo, Antonietta Gerarda Gravina, Erasmo Miele, Elena Cantone, Nicola Gennarelli, Rita Nocerino, Laura Carucci, Veronica Giglio, Felice Amato, Giuseppe Castaldo

**Affiliations:** ^1^Department of Translational Medical Science, University of Naples Federico II, Naples, Italy; ^2^CEINGE-Biotecnologie Avanzate s.c.ar.l., University of Naples Federico II, Naples, Italy; ^3^European Laboratory for the Investigation of Food-Induced Diseases, University of Naples Federico II, Naples, Italy; ^4^Task Force for Microbiome Studies, University of Naples Federico II, Naples, Italy; ^5^Department of Molecular Medicine and Medical Biotechnologies, University of Naples Federico II, Naples, Italy; ^6^Department of Woman, Child and General and Specialistic Surgery, University of Campania “Luigi Vanvitelli”, Naples, Italy; ^7^Division of Hepatogastroenterology, Department of Precision Medicine, University of Campania “Luigi Vanvitelli”, Naples, Italy; ^8^Department of Neuroscience, Reproductive and Odontostomatological Sciences, Ear, Nose and Throat (ENT) Section, University of Naples Federico II, Naples, Italy; ^9^Department of Clinical Medicine and Surgery, University of Naples Federico II, Naples, Italy

**Keywords:** COVID-19, angiotensin-converting enzyme 2, transmembrane serine protease-2, neuropilin-1, healthy subjects

## Abstract

**Background:** Clinical features of severe acute respiratory syndrome coronavirus 2 (SARS-CoV-2) infection seem to differ in children compared to that in adults. It has been hypothesized that the lower clinical severity in children could be influenced by differential expression of the main host functional receptor to SARS-CoV-2, the angiotensin-converting enzyme 2 (ACE2), but data are still conflicting. To explore the origin of age-dependent clinical features of coronavirus disease 2019 (COVID-19), we comparatively evaluated the expression in children and adult subjects of the most relevant mediators of the SARS-CoV-2 infection: ACE2, angiotensin-converting enzyme 1 (ACE1), transmembrane serine protease-2 (TMPRSS2), and neuropilin-1 (NRP1), at upper respiratory tract and small intestine level.

**Methods:** The expression of *ACE2, ACE1, TMPRSS2*, and *NRP1* in nasal epithelium and in small intestine epithelium was investigated by quantitative real-time PCR analysis.

**Results:** We found no differences in *ACE2, ACE1*, and *TMPRSS2* expression in the nasal epithelium comparing children and adult subjects. In contrast, nasal epithelium *NRP1* expression was lower in children compared to that in adults. Intestinal *ACE2* expression was higher in children compared to that in adults, whereas intestinal *ACE1* expression was higher in adults. Intestinal *TMPRSS2* and *NRP1* expression was similar comparing children and adult subjects.

**Conclusions:** The lower severity of SARS-CoV-2 infection observed in children may be due to a different expression of nasal *NRP1*, that promotes the virus interaction with ACE2. However, the common findings of intestinal symptoms in children could be due to a higher expression of *ACE2* at this level. The insights from these data will be useful in determining the treatment policies and preventive measures for COVID-19.

## Introduction

Throughout the world, fewer cases of coronavirus disease 2019 (COVID-19) have been reported in children than in adults. Most infected children appear to have a milder course, more frequent gastrointestinal symptoms and have better outcomes overall (1–8). Defining the mechanisms underlying this disease pattern maybe relevant for the development of effective public health strategies. It has been hypothesized that the lower risk among children could be influenced by differential expression of angiotensin-converting enzyme 2 (ACE2), which serves as the receptor used by SARS-CoV-2 for host entry (9, 10), but data on a possible age-dependent *ACE2* expression pattern are still conflicting (11–16). Additionally, data on other cellular components involved in SARS-CoV-2 infection remain largely elusive. The spike glycoprotein of SARS-CoV-2 is processed by transmembrane serine protease-2 (*TMPRSS2*) prior to membrane fusion. Neuropilin-1 (*NRP1*) acts as transmembrane receptor able to bind the C terminus of the S1 protein, generated by furin cleavage, termed the “C-end rule” (CendR). Thus, TMPRSS2 is essential to release the viral content into the host cells (12), whereas NRP1 plays a pivotal role in increasing the infectivity of SARS-CoV-2 (17, 18).

We conducted a prospective observational study to comparatively evaluate the expression of *ACE2, TMPRSS2*, and *NRP1* genes at two most relevant entry sites for SARS-CoV-2, the upper respiratory tract and the small intestine (9), in children and adult subjects. Angiotensin-converting enzyme 1 (*ACE1*) expression was also evaluated at both sites. ACE1 represents a positive regulator of the renin-angiotensin system (RAS) which promotes angiotensin II (AngII) conversion and subsequent vasoconstriction and inflammation (19). Inflammation is a key mechanism by which elevated Ang II signaling and ACE1/ACE2 imbalance cause cellular injury, aggravating the clinical severity of SARS-CoV-2 infection (20).

## Methods

### Study Population

From May 19th to July 30th 2020, individuals of both sexes, all Caucasian, aged 1–10 years in the pediatric population and 20–80 years in the adult population, were consecutively evaluated at tertiary centers for pediatrics, gastroenterology, and otolaryngology for suspected respiratory or gastrointestinal organic disorders. Emerging data suggested that children younger than 10 years have lower susceptibility to SARS-CoV-2 infection than adults, with adolescents appearing to have similar susceptibility to adults (21–23). These data have been also highlighted in a recent systematic review and meta-analysis (24). Thus, we planned to evaluate two populations with well-distinct age ranges: the pediatric populations composed by children and pre-adolescents aged between 1 and 10 years, and the adult population composed by subjects aged between 20 and 80 years.

The inclusion criteria were subjects of both sexes aged between 1 and 10 years for the pediatric population and between 20 and 80 years for the adult population, necessity of full clinical, laboratory, endoscopic and histologic evaluation because the presence of the following symptoms from at least 4 weeks: abdominal pain, constipation, diarrhea and cough, with negative results for serological and PCR tests for SARS-CoV-2.

The exclusion criteria were positive history for immunodeficiencies, allergies, metabolic and genetic disorders, tumors, cystic fibrosis, malformations, cardiovascular diseases, hypertension, inflammatory bowel diseases, adverse food reactions, celiac disease, autoimmune disorders, infectious diseases, malnutrition, or any drug use in the previous 12 weeks.

The clinical status of the study subjects was carefully assessed by a multidisciplinary team composed of gastroenterologists, otolaryngologists, pediatricians (experienced in pediatric allergy and gastroenterology), pediatric nurses, and dietitians. The diagnostic work up also included the evaluation of SARS-CoV-2 infection using molecular swab PCR test (Allplex 2019-nCoV assay, Seegene, Inc, Seul, South Korea) and anti-SARS-CoV-2 IgG and IgM serum levels by chemiluminescent immunoassay (iFlash-SARS-CoV-2; Shenzhen Yhlo Biotech Co. Ltd.). These evaluations were performed at enrolment and after 7 days. At the end of the diagnostic work up, only subjects with negative results for all diagnostic procedures were considered for the study. For all subjects included in the study, a subsequent visit performed by the multidisciplinary team 6 months after the enrolment was also planned to assess the persistence of well-being status.

### Sample Collection and Analysis

Nasal epithelial samples were collected using a cytology brush (EndoscanPlus, Medico, Melbourne, Australia), whereas small intestinal epithelial biopsies were collected during endoscopic procedures. The histologic features of all samples were evaluated by an experienced pathologist unaware of any relevant clinical information concerning the subjects. Samples were immediately placed in RNAlater (Thermo Fisher Scientific, Waltham, MA, USA) and stored at −80°C until analysis. Total RNA was extracted with the TRIzol reagent (Invitrogen, Thermo Scientific, Waltham, MA, USA). The samples were quantified using the NanoDrop 2000c spectrophotometer (Thermo Scientific) and RNA quality and integrity were assessed with the Experion RNA Standard Sense kit (Bio-Rad, Hercules, CA, USA). cDNA was synthesized with random primers using the SensiFASTcDNA Synthesis Kit (Bioline) on the CFX96 RealTime System instrument (Bio-Rad, Hercules, CA, USA). Quantitative real-time PCR (qRT-PCR) analysis was performed using the SensiFAST SYBR Hi-ROX Kit (Bioline) on the7900HT Fast Real-Time PCR System (Applied Biosystems) with the primers described in Table 1. Data analysis was performed using the comparative threshold cycle (CT) method and expressed as 2^∧^-delta CT (25). Gene expression was normalized against the expression of the reference gene hypoxanthine phosphoribosyltransferase 1 (*HPRT*).

**Table 1:** The primer sequences used for quantitative real-time PCR analysis.

**Gene**	**Sequence (5^**′**^-3^**′**^)**
*ACE2* (NM_021804.3)	GCAGACCAAAGCATCAAAGTG
	GGTTTCAAATTAGCCACTCGC
*ACE1* (NM_000789.4)	CAGAACACCACTATCAAGCG
	GTCTTCATATTTCCGGGACG
*TMPRSS2* (NM_005656.4)	AGCCTCTGACTTTCAACGAC
	TCAATGAGAAGCACCTTGGC
*NRP1*(NM_003873)	GCCACAGTGGAACAGGTGA
	ATGACCGTGGGCTTTTCTGT
*HPRT* (NM_000194.3)	GACCAGTCAACAGGGGACAT
	GTGTCAATTATATCTTCCACAATCAAG

### Ethics

The study protocol, the subject information sheet, the informed consent form, and the clinical chart were reviewed and approved by the Ethics Committee of the University of Naples Federico II (N.274/17/ESCOVID19; 18/05/2020). At the baseline, written and signed informed consent was obtained from all adult participants and from all parents/tutors of the minors. The study was conducted in accordance with the Helsinki Declaration (Fortaleza revision 2013), the Good Clinical Practice Standards (CPMP/ICH/135/95), and the current Decree-Law 196/2003 regarding personal data and all the requirements set out in the European regulations on this subject.

### Statistical Analysis

Descriptive statistics were reported as means and standard deviations (SD) for continuous variables. To evaluate the differences among continuous variables, the independent sample *t*-test was performed.

The level of significance for all statistical tests was 2-sided, *p* < 0.05. All data were collected in a dedicated database and analyzed by a statistician using GraphPad Prism 7 (La Jolla, CA, USA).

## Results

A total of 38 children and 35 adult subjects were evaluated for the study. Eight children and five adults were excluded because of the presence of allergies (*n* = 4), infections (*n* = 4), food intolerances (*n* = 3), celiac disease (*n* = 1), and inflammatory bowel disease (*n* = 1). Thus, 30 children and 30 adult subjects were finally considered for the enrolment.

Among 15 children who underwent to endoscopic procedure, nine complained of abdominal pain and diarrhea, one abdominal pain and constipation, and five diarrhea. Among 15 adult subjects who underwent to endoscopic procedure, twelve complained of abdominal pain and diarrhea, and three diarrhea.

All these subjects presented negative results of the diagnostic work up, including endoscopic and histologic evaluation, peripheral cell blood count, transaminases, inflammatory biomarkers and COVID-19 screening tests. None of these subjects presented organic conditions and/or COVID-19 infection during the 6 months follow up after sampling. Main demographic and clinical features of the study population are summarized in the Table 2.

**Table 2:** Main demographic and clinical features of the study population.

	**Pediatric subjects**		**Adult subjects**	
	**Nasal brushing**	**Small intestine biopsy**	**Nasal brushing**	**Small intestine biopsy**
*N*	15	15	15	15
Male, *n* (%)	9 (60)	7 (46.6)	6 (40)	9 (60)
Ethnicity, Caucasian, *n* (%)	15 (100)	15 (100)	15 (100)	15 (100)
Median age, years (range)	1 (1–3)	8.5 (1–10)	29 (20–55)	59 (22–80)
**SARS-CoV-2 PCR**				
- Positive (%)	0 (0)	0 (0)	0 (0)	0 (0)
- Negative (%)	15 (100)	15 (100)	15 (100)	15 (100)
**IgM against SARS-CoV-2**				
- Positive ( ≤ 1 kU/L %)	0 (0)	0 (0)	0 (0)	0 (0)
- Negative (> 10 kU/L; %)	15 (100)	15 (100)	15 (100)	15 (100)
**IgG against SARS-CoV-2**				
- Positive (> 30 kAU/L; %)	0 (0)	0 (0)	0 (0)	0 (0)
- Negative ( ≤ 10 kAU/L;%)	15 (100)	15 (100)	15 (100)	15 (100)
White blood cells count (x10^3^ cell/μL)^*^	9.7 (8.5–10.6)	8.6 (7.5–9.3)	8.5 (5–10.5)	7.7 (4.9–10.8)
Hemoglobin (g/dL)^*^	13.3 (12.6–14.5)	13.3 (12.5–14.0)	14.0 (12.1–16.0)	13.5 (12.3–15.7)
Red blood cells count (x10^6^ cell/μL)^*^	4.7 (4.1–5.2)	4.6 (4.0–5.1)	4.7 (4.3–5.2)	5.0 (4.5–5.4)
C-reactive protein (mg/dL)^*^	0.3 (0–0.4)	0.1 (0–0.3)	0.2 (0.1–0.5)	0.2 (0.1–0.4)
Erythrocyte sedimentation rate (mm)^*^	7 (0–10)	7 (3–12)	8 (0–13)	8 (0–15)
Creatinine (mg/dL)^*^	0.8 (0.7–1.2)	1.0 (0.8–1.2)	0.9 (0.7–1.2)	0.8 (0.7–1.1)
Aspartate aminotransferase (U/l)^*^	16 (12–32)	19 (16–22)	20 (15–40)	21 (18–42)
Alanine aminotransferase (U/l)^*^	11 (8–22)	12 (8–21)	20 (12–44)	20 (13–44)
**Endoscopic findings**, ***n*** **(%)**				
- Normal	15 (100)	15 (100)	15 (100)	15 (100)
**Histologic findings**, ***n*** **(%)**				
- Normal	15 (100)	15 (100)	15 (100)	15 (100)

* *All values were reported as median with range (min-max)*.

**Figure 1:**
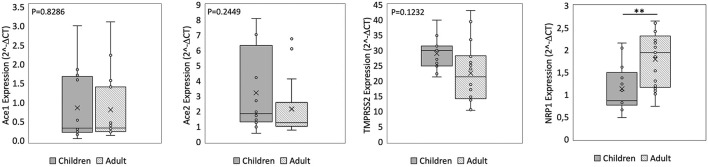
qPCR analysis of Angiotensin I Converting Enzyme (*ACE1*), Angiotensin II Converting Enzyme (*ACE2*), Transmembrane Serine Protease 2 (*TMPRSS2*), and Neuropilin-1 *(NRP1*) genes in nasal epithelium from children and adult subjects. Comparative expression of *ACE1, ACE2, TMPRSS2*, and *NRP1* in nasal epithelium of children (*n* = 15) and adult subjects (*n* = 15). Data analysis was performed using the comparative threshold cycle (CT) method and expressed as 2^∧^-delta CT. Gene expression was normalized against the expression of the reference gene hypoxanthine phosphoribosyltransferase 1 (*HPRT*). Data are expressed as median ± SD, the X in the bars indicates mean values. *P*-value are reported in the graphs; significant differences are indicated as ^*^p < 0.01; ^**^p < 0.005.

**Figure 2:**
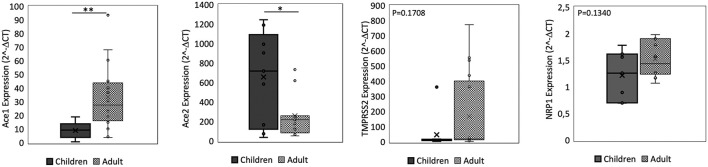
qPCR analysis of Angiotensin I Converting Enzyme (*ACE1*), Angiotensin II Converting Enzyme (*ACE2*), Transmembrane Serine Protease 2 (*TMPRSS2*), and Neuropilin-1 (*NRP1*) genes in small intestine from children and adult subjects. Comparative expression of *ACE1, ACE2, TMPRSS2*, and *NRP1* in small intestine of children (*n* = 15) and adult subjects (*n* = 15). Data analysis was performed using the comparative threshold cycle (CT) method and expressed as 2^∧^-delta CT. Gene expression was normalized against the expression of the reference gene hypoxanthine phosphoribosyltransferase 1 (*HPRT*). Data are expressed as median ± SD, the X in the bars indicates mean values. *P*-value are reported in the graphs; significant differences are indicated as ^*^p < 0.01; ^**^p < 0.005.

We found no significant difference in *ACE2* and *ACE1* expression in the nasal epithelium comparing children and adult subjects. A similar expression pattern was also observed for *TMPRSS2*. In contrast, the *NRP1* expression in the nasal epithelium resulted 1.6 fold-time higher in adults than in children (*p* = 0.00045) (Figure 1). In the small intestine, we observed that *ACE2* expression was 2.5 fold-time higher in children compared to that observed in adults, whereas the *ACE1* expression was 3.7 fold-time higher in adults than in children. Lastly, similar intestinal expression of *TMPRSS2* and *NRP1* was observed in the two study populations (Figure 2).

Comparing the expression of these SARS-CoV-2 mediators at two different sites, the *ACE2* and *ACE1* expression levels were respectively 120–200 fold-time and 15–30 fold-time higher at intestinal level compared to that observed in nasal epithelium. Small intestinal *TMPRSS2* expression was 2 fold-time higher if compared to that observed in nasal epithelium. *NRP1* expression was similar at the two sites (Figures 1, 2).

## Discussion

It has been hypothesized that differential expression of *ACE2* could be responsible for the general lower severity of SARS-CoV-2 infection in children, but data are still conflicting (3–6, 26). In this study, we focused on the expression of the molecular mediators that regulate the SARS-CoV-2 entry into host cells: the *ACE2* receptor, that acts as molecular doorway to the virus; the serine protease *TMPRSS2*, that is employed by SARS-CoV-2 for spike protein priming (9–14); and the *NRP1*, a SARS-CoV-2 co-receptor, that enhances the ability of SARS-CoV-2 to enter and potentiates the infection in host cells (17, 18).

We observed similar *ACE2* and *TMPRSS2* expression in the upper respiratory tract in children and adult subjects. On the contrary, the *NRP1* expression at this level resulted significantly lower in children. This data is well in line with recent evidence suggesting a different expression of *NRP1* in neonates and adults (27). The relevance of this finding is well-supported by recent evidence suggesting an important role exerted by NRP1 in driving the clinical severity of COVID-19 (17, 18).

In agreement with previous data (28–30), we found that *ACE2* expression in the small intestine was more than 100 times higher than that in the respiratory epithelium. This finding supports the concept that the gut could be an active site for SARS-CoV-2 replication (31). However, its expression was more abundant in the small intestine of children compared to that in adult subjects. In contrast, *ACE1* expression was lower in children, and intestinal *TMPRSS2* and *NRP1* expression was similar in the two study populations. Apparently these data are in contrast with what observed in small intestinal specimens collected from patients affected by inflammatory bowel diseases where similar *ACE2* and *TMPRSS2* expression was reported comparing pediatric vs. adult subjects (14), or in tissue specimens collected from allergic patients (5) or with data deriving from the analysis of public gene expression datasets (32). It should be considered that these data derived mainly from subjects affected by inflammatory conditions, whereas the strength of our study is the evaluation of a well-characterized population of subjects free of any organic conditions, in which we evaluated the main mediators of SARS-CoV-2 infection.

The main limitations of our study are the relatively low number of observations, the absence of subjects between the ages of 10 and 20 or older than 80 years and the absence of data concerning other potential co-factors involved in the COVID-19 pathogenesis.

The results obtained in this study suggest that the lower severity of SARS-CoV-2 infection observed in children may be due to a different expression in the respiratory tract of *NRP1*, the pivotal co-receptor that enhance SARS-CoV-2 entry into the host cells (17, 18). This data also support the potential role of NRP1 as therapeutic target against COVID-19, as suggested by others (33).

It has been observed that gastrointestinal symptoms could be more frequent in pediatric COVID-19 cases (1, 2, 5, 34). These clinical features could be due to a different expression of *ACE2* and *ACE1* in the small intestine of children. In fact, the combination of higher *ACE1* with lower *ACE2* expression may facilitate angiotensin II (AngII)-mediated vasoconstriction, inflammation and fibrosis, thereby aggravating the severity of SARS-CoV-2-induced gastrointestinal symptoms (20, 35, 36).

Other co-factors may confer protection against SARS-CoV-2 in children, including cross-reactive humoral and T-cell immunity between common coronaviruses and SARS-CoV-2, protective Th2 immunity, different reactivity of innate immunity response, and lower production of inflammatory cytokines (7, 37, 38). Emerging evidence suggest also a role of anti-oxidant nuclear factor (erythroid –derived-2)-like 2 (Nrf-2) -interacting foods, genetic predisposition and gut microbiota composition and function to regulate the immune system response in patients with COVID-19 (39–41). Additionally, higher prevalence of comorbidities in adults, as compared to children, could also contribute to different clinical features and outcomes.

In conclusion, our study suggests a difference in the *NRP1* and of *ACE2/ACE1* expression between pediatric and adult populations, which may contribute to explain the different age-related clinical patterns of SARS-CoV-2 infection. The better definition of all these co-factors will be useful in determining future treatment policies and preventive measures against COVID-19.

## Data Availability Statement

The raw data supporting the conclusions of this article will be made available by the authors, without undue reservation.

## Ethics Statement

The study protocol, the subject information sheet, the informed consent form, and the clinical chart were reviewed and approved by the Ethics Committee of our University of Naples Federico II Institution. Written informed consent to participate in this study was provided by the participants' legal guardian/next of kin.

## Author Contributions

RB, FA, and GCa designed the study, coordinated the research team, and wrote the first draft of this report. CS, AG, EM, EC, NG, LC, and VG were responsible for the study subjects and evaluated their health status. FA, LP, MC, GCe, CB, and IZ conducted the laboratory experiments. FA and LP performed the statistical analysis and data interpretation. All of the authors revised and approved the final version of this article.

## Conflict of Interest

The authors declare that the research was conducted in the absence of any commercial or financial relationships that could be construed as a potential conflict of interest.

## Publisher's Note

All claims expressed in this article are solely those of the authors and do not necessarily represent those of their affiliated organizations, or those of the publisher, the editors and the reviewers. Any product that may be evaluated in this article, or claim that may be made by its manufacturer, is not guaranteed or endorsed by the publisher.

## References

[1] Wang JG Cui HR Tang HB Deng XL. Gastrointestinal symptoms and fecal nucleic acid testing of children with 2019 coronavirus disease: a systematic review and meta-analysis. Sci Rep. (2020) 10:17846. 10.1038/s41598-020-74913-0 33082472 PMC7576139

[2] Parasa S Desai M Thoguluva V Patel HK Kennedy KF Roesch T. Prevalence of gastrointestinal symptoms and fecal viral shedding in patients with coronavirus disease 2019: a systematic review and meta-analysis. J Am Med Assoc Netw Open. (2020) 3:e2011335. 10.1001/jamanetworkopen.2020.11335 32525549 PMC7290409

[3] de Souza TH Nadal JA Nogueira RJN Pereira RM Brandão MB. Clinical manifestations of children with COVID-19: a systematic review. Pediatr Pulmonol. (2020) 55:1892–9. 10.1002/ppul.24885 32492251 PMC7300659

[4] Mantovani A Rinaldi E Zusi C Beatrice G Saccomani MD Dalbeni A. Coronavirus disease 2019 (COVID-19) in children and/or adolescents: a meta-analysis. Pediatr Res. (2021) 89:733–7. 10.1038/s41390-020-1015-2 32555539

[5] Mansourian M Ghandi Y Habibi D Mehrabi S. COVID-19 infection in children: a systematic review and meta-analysis of clinical features and laboratory findings. Arch Pediatr. (2021) 28:242–8. 10.1016/j.arcped.2020.12.008 33483192 PMC7794595

[6] Cui X Zhao Z Zhang T Guo W Guo W Zheng J. A systematic review and meta-analysis of children with coronavirus disease 2019 (COVID-19). J Med Virol. (2020) 93:1057–69. 10.1002/jmv.26398 32761898 PMC7436402

[7] Ding Y Yan H Guo W. Clinical characteristics of children with COVID-19: a meta-analysis. Front Pediatr. (2020) 8:431. 10.3389/fped.2020.00431 32719759 PMC7350605

[8] Badal S Thapa Bajgain K Badal S Thapa R Bajgain BB Santana MJ. Prevalence, clinical characteristics, and outcomes of pediatric COVID-19: a systematic review and meta-analysis. J Clin Virol. (2021) 135:104715. 10.1016/j.jcv.2020.104715 33348220 PMC7723460

[9] Hoffmann M Kleine-Weber H Schroeder S Krüger N Herrler T Erichsen S. SARS-CoV-2 cell entry depends on ACE2 and TMPRSS2 and is blocked by a clinically proven protease inhibitor. Cell. (2020) 181:271–80.e8. 10.1016/j.cell.2020.02.052 32142651 PMC7102627

[10] Shang J Wan Y Luo C Ye G Geng Q Auerbach A. Cell entry mechanisms of SARS-CoV-2. Proc Natl Acad Sci USA. (2020) 117:11727–34. 10.1073/pnas.2003138117 32376634 PMC7260975

[11] Bunyavanich S Vicencio A. Nasal gene expression of angiotensin-converting enzyme 2 in children and adults. J Am Med Assoc. (2020) 323:2427–9. 10.1001/jama.2020.8707 32432657 PMC7240631

[12] Suárez-Fariñas M Tokuyama M Wei G Huang R Livanos A Jha D. Intestinal inflammation modulates the expression of ACE2 and TMPRSS2 and potentially overlaps with the pathogenesis of SARS-CoV-2-related disease. Gastroenterology. (2021) 160:287–301.e20. 10.1101/2020.05.21.109124 32980345 PMC7516468

[13] Saheb Sharif-Askari N Saheb Sharif-Askari F Alabed M Temsah MH Al Heialy S Hamid Q. Airways expression of SARS-CoV-2 receptor, ACE2, and TMPRSS2 is lower in children than adults and increases with smoking and COPD. Mol Ther Methods Clin Dev. (2020) 18:1–6. 10.1016/j.omtm.2020.05.013 32537478 PMC7242205

[14] Simon AK Hollander GA McMichael A. Evolution of the immune system in humans from infancy to old age. Proc Biol Sci. (2015) 282:20143085. 10.1098/rspb.2014.3085 26702035 PMC4707740

[15] Pandit K Gupta S Sharma AG. Clinico-pathogenesis of COVID-19 in children. Indian J Biochem Biophys. (2020) 57:264–9.

[16] Kaseb AO Mohamed YI Malek AE Raad II Altameemi L Li D. The impact of angiotensin-converting enzyme 2 (ACE2) expression on the incidence and severity of COVID-19 infection. Pathogens. (2021) 10:379. 10.3390/pathogens10030379 33809851 PMC8004186

[17] Cantuti-Castelvetri L Ojha R Pedro LD Djannatian M Franz J Kuivanen S. Neuropilin-1 facilitates SARS-CoV-2 cell entry and infectivity. Science. (2020) 370:856–60. 10.1126/science.abd2985 33082293 PMC7857391

[18] Daly JL Simonetti B Klein K Chen KE Williamson MK Antón-Plágaro C. Neuropilin-1 is a host factor for SARS-CoV-2 infection. Science. (2020) 370:861–5. 10.1126/science.abd3072 33082294 PMC7612957

[19] Alexandre J Cracowski JL Richard V Bouhanick B. 'Drugs, COVID-19' working group of the French Society of Pharmacology, Therapeutics. Renin-angiotensin-aldosterone system and COVID-19 infection. Ann Endocrinol. (2020) 81:63–7. 10.1016/j.ando.2020.04.005 PMC7172808 32370986

[20] Sriram K Insel PAA. Hypothesis for pathobiology and treatment of COVID-19: the centrality of ACE1/ACE2 imbalance. Br J Pharmacol. (2020) 177:4825–44. 10.1111/bph.15082 32333398 PMC7572451

[21] Park YJ Choe YJ Park O Park SY Kim YM Kim J. COVID-19 National Emergency Response Center, Epidemiology and Case Management Team. Contact tracing during coronavirus disease outbreak, South Korea, 2020. Emerg Infect Dis. (2020) 26:2465–8. 10.3201/eid2610.201315 32673193 PMC7510731

[22] Kim J Choe YJ Lee J Park YJ Park O Han MS. Role of children in household transmission of COVID-19. Arch Dis Child. (2020) 106:709–11. 10.1136/archdischild-2020-319910 32769089

[23] Li X Xu W Dozier M He Y Kirolos A Theodoratou E. The role of children in transmission of SARS-CoV-2: a rapid review. J Glob Health. (2020) 10:011101. 10.7189/jogh.10.011101 32612817 PMC7323934

[24] Viner RM. Mytton, OT; Bonell C, Melendez-Torres GJ, Ward J, Hudson L, et al. Susceptibility to SARS-CoV-2 infection among children and adolescents compared with adults: a systematic review and meta-analysis. J Am Med Assoc Pediatr. (2021) 175:143–56. 10.1001/jamapediatrics.2020.4573 32975552 PMC7519436

[25] Livak KJ Schmittgen TD. Analysis of relative gene expression data using real time quantitative PCR and the 2 (-Delta Delta C(T)) method. Methods. (2001) 25:402–8. 10.1006/meth.2001.1262 11846609

[26] Chen J Jiang Q Xia X Liu K Yu Z Tao W. Individual variation of the SARS-CoV-2 receptor ACE2 gene expression and regulation. Aging Cell. (2020) 19:e13168. 10.1111/acel.13168 32558150 PMC7323071

[27] Heinonen S Helve O Andersson S Janér C Süvari L Kaskinen A. Nasal expression of SARS-CoV-2 entry receptors in newborns. Archiv Dis Childh. (2021) 2020:321334. 10.1136/archdischild-2020-321334 33990387

[28] Xie X Chen J Wang X Zhang F Liu Y. Age- and gender-related difference of ACE2 expression in rat lung. Life Sci. (2006) 78:2166–71. 10.1016/j.lfs.2005.09.038 16303146 PMC7094566

[29] Yoon HE Kim EN Kim MY Lim JH Jang IA Ban TH. Age-associated changes in the vascular renin-angiotensin system in mice. Oxid Med Cell Longev. (2016) 2016:6731093. 10.1155/2016/6731093 27200147 PMC4855022

[30] Xu H Zhong L Deng J Peng J Dan H Zeng X. High expression of ACE2 receptor of 2019-nCoV on the epithelial cells of oral mucosa. Int J Oral Sci. (2020) 12:8. 10.1038/s41368-020-0074-x 32094336 PMC7039956

[31] Li MY Li L Zhang Y Wang XS. Expression of the SARS-CoV-2 cell receptor gene ACE2 in a wide variety of human tissues. Infect Dis Poverty. (2020) 9:45. 10.1186/s40249-020-00662-x 32345362 PMC7186534

[32] Devaux CA Lagier JC Raoult D. New insights into the physiopathology of COVID-19: SARS-CoV-2-associated gastrointestinal illness. Front Med. (2021) 8:640073. 10.3389/fmed.2021.640073 33681266 PMC7930624

[33] Klaewkla M Charoenwongpaiboon T Mahalapbutr P. Molecular basis of the new COVID-19 target neuropilin-1 in complex with SARS-CoV-2 S1 C-end rule peptide and small-molecule antagonists. J Mol Liq. (2021) 335:116537. 10.1016/j.molliq.2021.116537 34031621 PMC8133821

[34] Lukassen S Chua RL Trefzer T Kahn NC Schneider MA Muley T. SARS-CoV-2 receptor ACE2 and TMPRSS2 are primarily expressed in bronchial transient secretory cells. EMBO J. (2020) 39:e105114. 10.15252/embj.2020105114 32246845 PMC7232010

[35] Giacomet V Barcellini L Stracuzzi M Longoni E Folgori L Leone A. COVID-19 Pediatric network. Gastrointestinal symptoms in severe COVID-19 children. Pediatr Infect Dis J. (2020) 39:e317–20. 10.1097/INF.0000000000002843 32932333

[36] Behl T Kaur I Bungau S Kumar A Uddin MS Kumar C. The dual impact of ACE2 in COVID-19 and ironical actions in geriatrics and pediatrics with possible therapeutic solutions. Life Sci. (2020) 257:118075. 10.1016/j.lfs.2020.118075 32653522 PMC7347488

[37] Steinman JB Lum FM Pui-Kay Ho P Kaminski N Steinman L. Reduced development of COVID-19 in children reveals molecular checkpoints gating pathogenesis illuminating potential therapeutics. Proc Natl Acad Sci USA. (2020) 117:24620–6. 10.1073/pnas.2012358117 32883878 PMC7547272

[38] Castagnoli R Votto M Licari A Brambilla I Bruno R Perlini S. Severe acute respiratory syndrome coronavirus 2 (SARS-CoV-2) infection in children and adolescents a systematic review. J Am Med Assoc Pediatr. (2020) 2:1–8. 10.1001/jamapediatrics.2020.1467 32320004

[39] Bousquet J Cristol JP Czarlewski W Anto JM Martineau A Haahtela T. Nrf2-interacting nutrients and COVID-19: time for research to develop adaptation strategies. Clin Transl Allergy. (2020) 10:58. 10.1186/s13601-020-00362-7 33292691 PMC7711617

[40] Pairo-Castineira E Clohisey S Klaric L Bretherick AD Rawlik K Pasko D. Genetic mechanisms of critical illness in COVID-19. Nature. (2021) 591:92–8. 10.1038/s41586-020-03065-y 33307546

[41] Yeoh YK Zuo T Lui GC Zhang F Liu Q Li AY. Gut microbiota composition reflects disease severity and dysfunctional immune responses in patients with COVID-19. Gut. (2021) 70:698–706. 10.1136/gutjnl-2020-323020 33431578 PMC7804842

