# Corrigendum: Age-Related Differences in the Expression of Most Relevant Mediators of SARS-CoV-2 Infection in Human Respiratory and Gastrointestinal Tract

**DOI:** 10.3389/fped.2021.790285

**Published:** 2021-11-10

**Authors:** Roberto Berni Canani, Marika Comegna, Lorella Paparo, Gustavo Cernera, Cristina Bruno, Caterina Strisciuglio, Immacolata Zollo, Antonietta Gerarda Gravina, Erasmo Miele, Elena Cantone, Nicola Gennarelli, Rita Nocerino, Laura Carucci, Veronica Giglio, Felice Amato, Giuseppe Castaldo

**Affiliations:** ^1^Department of Translational Medical Science, University of Naples Federico II, Naples, Italy; ^2^CEINGE-Biotecnologie Avanzate s.c.ar.l., University of Naples Federico II, Naples, Italy; ^3^European Laboratory for the Investigation of Food-Induced Diseases, University of Naples Federico II, Naples, Italy; ^4^Task Force for Microbiome Studies, University of Naples Federico II, Naples, Italy; ^5^Department of Molecular Medicine and Medical Biotechnologies, University of Naples Federico II, Naples, Italy; ^6^Department of Woman, Child and General and Specialistic Surgery, University of Campania “Luigi Vanvitelli”, Naples, Italy; ^7^Division of Hepatogastroenterology, Department of Precision Medicine, University of Campania “Luigi Vanvitelli”, Naples, Italy; ^8^Department of Neuroscience, Reproductive and Odontostomatological Sciences, Ear, Nose and Throat (ENT) Section, University of Naples Federico II, Naples, Italy; ^9^Department of Clinical Medicine and Surgery, University of Naples Federico II, Naples, Italy

**Keywords:** COVID-19, angiotensin-converting enzyme 2, transmembrane serine protease-2, neuropilin-1, healthy subjects

In the original article, there was a mistake in the order of Figures 1 and 2 as published. The figures are given in the correct order below.

**Figure 1:**
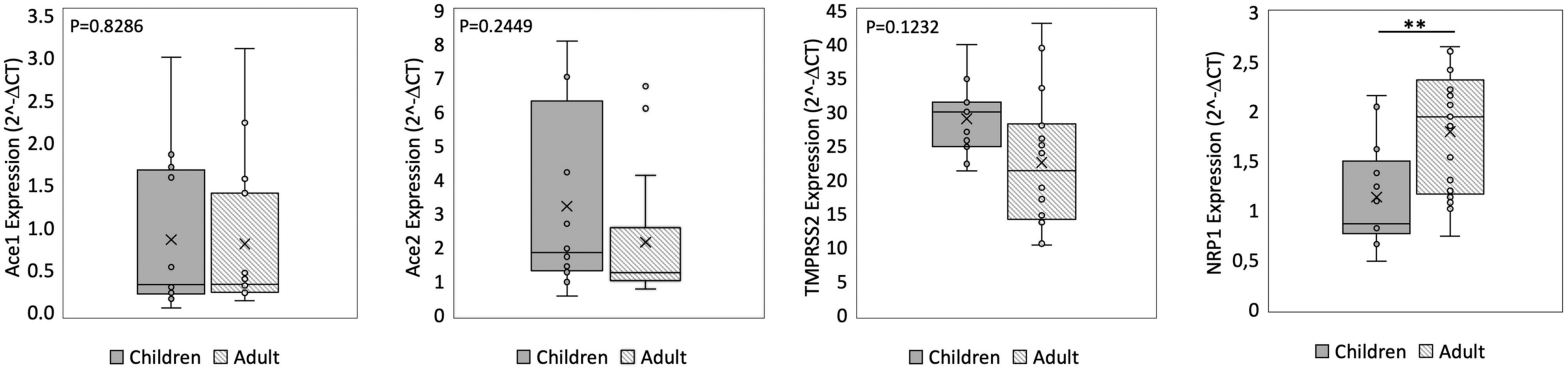
qPCR analysis of Angiotensin I Converting Enzyme (*ACE1*), Angiotensin II Converting Enzyme (*ACE2*), Transmembrane Serine Protease 2 (*TMPRSS2*), and Neuropilin-1 *(NRP1*) genes in nasal epithelium from children and adult subjects. Comparative expression of *ACE1, ACE2, TMPRSS2*, and *NRP1* in nasal epithelium of children (*n* = 15) and adult subjects (*n* = 15). Data analysis was performed using the comparative threshold cycle (CT) method and expressed as 2^∧^-delta CT. Gene expression was normalized against the expression of the reference gene hypoxanthine phosphoribosyltransferase 1 (*HPRT*). Data are expressed as median ± SD, the X in the bars indicates mean values. *P*-value are reported in the graphs; significant differences are indicated as ^*^p < 0.01; ^**^p < 0.005.

**Figure 2:**
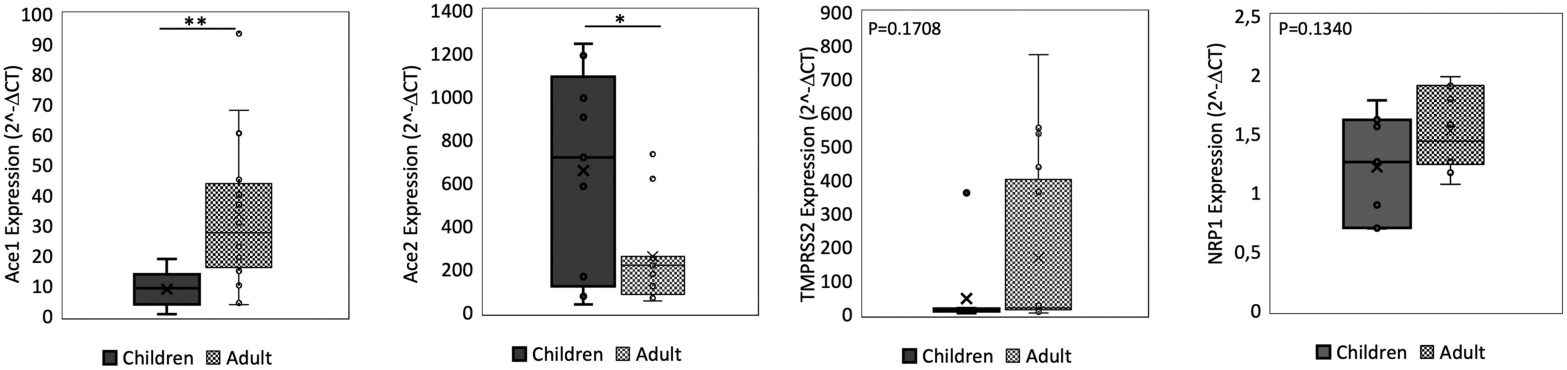
qPCR analysis of Angiotensin I Converting Enzyme (*ACE1*), Angiotensin II Converting Enzyme (*ACE2*), Transmembrane Serine Protease 2 (*TMPRSS2*), and Neuropilin-1 (*NRP1*) genes in small intestine from children and adult subjects. Comparative expression of *ACE1, ACE2, TMPRSS2*, and *NRP1* in small intestine of children (*n* = 15) and adult subjects (*n* = 15). Data analysis was performed using the comparative threshold cycle (CT) method and expressed as 2^∧^-delta CT. Gene expression was normalized against the expression of the reference gene hypoxanthine phosphoribosyltransferase 1 (*HPRT*). Data are expressed as median ± SD, the X in the bars indicates mean values. *P*-value are reported in the graphs; significant differences are indicated as ^*^p < 0.01; ^**^p < 0.005.

The authors apologize for the error and state that this does not change the scientific conclusions of the article in any way. The original article has been updated.

## Publisher's Note

All claims expressed in this article are solely those of the authors and do not necessarily represent those of their affiliated organizations, or those of the publisher, the editors and the reviewers. Any product that may be evaluated in this article, or claim that may be made by its manufacturer, is not guaranteed or endorsed by the publisher.

